# The Salutary Influence of Forest Bathing on Elderly Patients with Chronic Heart Failure

**DOI:** 10.3390/ijerph14040368

**Published:** 2017-03-31

**Authors:** Genxiang Mao, Yongbao Cao, Bozhong Wang, Sanying Wang, Zhuomei Chen, Jirong Wang, Wenmin Xing, Xiaoxu Ren, Xiaoling Lv, Jianhua Dong, Shasha Chen, Xiuyuan Chen, Guofu Wang, Jing Yan

**Affiliations:** 1 Zhejiang Provincial Key Laboratory of Geriatrics & Geriatrics Institute of Zhejiang Province, Zhejiang Hospital, No.12 Lingyin Road, Hangzhou 310013, China; maogenxiang@163.com (G.M.); hzzycyb@163.com (Y.C.); zjxzkf5200@sina.com (B.W.); sanyingwang309@126.com (S.W.); wangjr@zju.edu.cn (J.W.); xing-wenmin@hotmail.com (W.X.); jk4991@126.com (X.L.); chenhntx@163.com (S.C.); 2 Zhejiang Forestry Academy, Hangzhou 310023, China; zhuomeichen@163.com; 3 Hangzhou Forestry Academy, Hangzhou 310022, China; renxiaoxu2014@163.com (X.R.); jianhuadong@126.com (J.D.); 4 Forestry Bureau of Pan’an County, Jinhua 322300, China; hero7948@163.com

**Keywords:** chronic heart failure, forest bathing, inflammation, PM_2.5_

## Abstract

The aim of the current study was to test the hypothesis that forest bathing would be beneficial for elderly patients with chronic heart failure (CHF) as an adjunctive therapy. Two groups of participants with CHF were simultaneously sent to the forest or an urban control area for a four-day trip, respectively. Subjects exposed to the forest site showed a significant reduction of brain natriuretic peptide (BNP) in comparison to that of the city group and their own baseline levels. The values for the cardiovascular disease related pathological factors, including endothelin-1 (ET-1), and constituents of the renin-angiotensin system (RAS), including renin, angiotensinogen (AGT), angiotensin II (ANGII), and ANGII receptor type 1 or 2 (AT1 or AT2) in subjects exposed to the forest environment were lower than those in the urban control group. Obviously, a decreased level of inflammatory cytokines and improved antioxidant function was observed in the forest group rather than in the city group. The assessment of the profile of mood states (POMS) indicated that the negative emotional mood state was alleviated after forest bathing. As anticipated, a better air quality in the forest site was observed according to the detection of PM_2.5_ (particulate matter <2.5 μm) and negative ions. These results provided direct evidence that forest bathing has a beneficial effect on CHF patients, and thus may pave the way for potential development of forest bathing as an effective adjunctive therapy on cardiovascular disorders.

## 1. Introduction

Nowadays, many developing countries are in the midst of an epidemiological transition as the disease burden rapidly shifts from diseases related to nutritional deficiencies and infections to degenerative chronic diseases observed in the older population [1]. In China, cardiovascular diseases (CAD) accounted for more than 40% of the deaths of the residents in 2014, known as the predominant cause of mortality. It was estimated that the morbidity and patients of CAD will continue to increase in the next several decades due to the fast-growing older population and urbanization in China, which will cause an extremely serious burden for the public health. Chronic heart failure (CHF), a clinical syndrome that develops as a consequence of various cardiac diseases, such as coronary artery disease, either alone or in combination with hypertension, remains a rising global cause of morbidity and mortality [2,3]. As of 2011, it was estimated that there were 5.7 million individuals living with HF diagnosed in the USA alone [4]. On the other hand, there is emerging evidence linking long-term environmental pollution to cardiovascular diseases, e.g., CHF, in urban communities according to a series of major epidemiologic and observational studies [5,6,7]. As urbanization has become a prevailing trend all around the world today, urban air pollution, such as “haze weather” and ambient PM_2.5_ (particulate matter), are serious environmental problems in many developing countries. In China, the average concentration of PM_2.5_ was 47 μg/m^3^ in 2016 [8], higher than the safety standards of other countries, e.g., 47 μg/m^3^ in the US. This situation seems to be worse in crowded cities. It was reported that higher levels of PM_2.5_ were deleterious for human health, such as shortened life expectancy or high symptoms of anxiety [9,10]. Thus, better methods which can promote health conditions for those living in cities are of great significance. Additionally, currently available pharmacological therapies provide limited impact on the long-term outlook for patients with CHF. Moreover, it is hard to overcome the adverse effect of polypharmacy treatment against CHF as the etiology of CHF is complicated and treatment with a single drug is rarely used. Thus, it is necessary to develop novel therapeutic interventional strategies for CHF patients. Forest bathing or forest therapy, also known as *Shinrin-yoku* in Japan or forest healing in South Korea, is a re-immersion in the sensorium of the healthy forest which is similar tonatural aromatherapy [11]. Additionally, it seems to be in part like art therapy, which is a relatively young therapeutic discipline by using the creative process of art making to improve the mental, emotional, and even physical conditions of individuals, such as cancer patients [12]. Recently, in Asian countries, such as China and Japan, forest bathing has received increasing attention due to its health-promoting effects, including enhancing immune functions and decreasing blood pressure in hypertension patients, as well as stress relief effects. Specifically, South Korea is a leading country applying forest therapy, e.g., the official designation of forests for human health, and quite a large number of studies about forest therapy on human health have been published from South Korea, such as providing physical relaxation, soothing anxiety, and even relieving pain in individuals with chronic widespread pain [13,14]. Our previous work indicated that forest bathing lowered the circulating endothelin-1 (ET-1) level in normal young subjects or old patients with hypertension [15,16], which is known as one of the most potent vasoconstrictors and is always recognized as a stimulator on cardiovascular diseases [17]. Our recent studies also indicated the healthy effect of forest bathing on elderly patients with chronic obstructive pulmonary disease (COPD) [18]. Thus, it is well speculated that forest bathing would be salutary to CAD patients, such as chronic heart failure, with potential therapeutic benefits. To test our hypothesis, herein we investigated the effect of forest bathing on physiological and psychological responses of CHF patients. We chose a forest site in Pan’an County, Zhejiang Province, China, where the forest occupies 80.3% of the land area. Two groups of CHF patients were sent to the forest site or to an urban control area for a four-day trip, respectively. Blood was sampled before and after the experiment to evaluate the changes of HF- and CAD-related biological indicators. A profile of mood states (POMS) evaluation was used to assess changes in mood states. Our findings indicated that forest bathing resulted in decreases of brain natriuretic peptide (BNP), renin-angiotensin system (RAS), inflammation, and oxidative stress, which suggested a favorable effect on cardiovascular disorders as an adjuvant therapy.

## 2. Materials and Methods

### 2.1. Study Subjects

This study recruited 45 CHF patients as subjects from the urban area in Hangzhou City. Participants who met the following criteria were included: (1) patients with diagnosed CHF; (2) aged from 65 to 80 years; (3) class I–III cardiac function according to the criteria of the American New York Heart Association; (4) blood pressure (BP), with or without medical intervention, less than 150/90 mmHg; and (5) capable of taking care of themselves in daily life. The exclusion criteria are: (1) catching cold or suffering other acute diseases two weeks prior to the trial or during the trial process; (2) chronic history, including cancer, serious liver, kidney, brain, heart, lung diseases, etc.; (3) acute myocardial infarction or cerebrovascular accident within six months; and (4) experienced a severe trauma or a major surgery.

A total of 36 patients with CHF meeting the criteria as described above were enrolled as participants in this study. They were divided randomly at a ratio of 2:1 into two groups consisting of 24 people in forest group and 12 in city control group before the experiment. One participant in the forest group and two in city group quit during the experiment process, so there were finally 23 participants in the forest group and 10 in the city control group. Clinical characteristics of the participants are shown in Table 1. There was no statistical difference on indicators of baselines between the two groups in terms of age, body mass index, blood pressure, pulse, and the distribution of I–III cardiac function (%).

The study was approved by the ethics committee of Zhejiang Hospital, and the procedures were in accordance with the Helsinki Declaration of 1975 as revised in 1983. The study was fully explained to all the participants in both spoken and written form, specifically focusing on its purpose, the precise procedures that would be used, and any possible adverse events. Signed informed consent was obtained from every subject. The trial was registered in Chinese Clinical Trial Registry (ChiCTR-OOC-15006853).

### 2.2. Study Design

**Figure 1:**
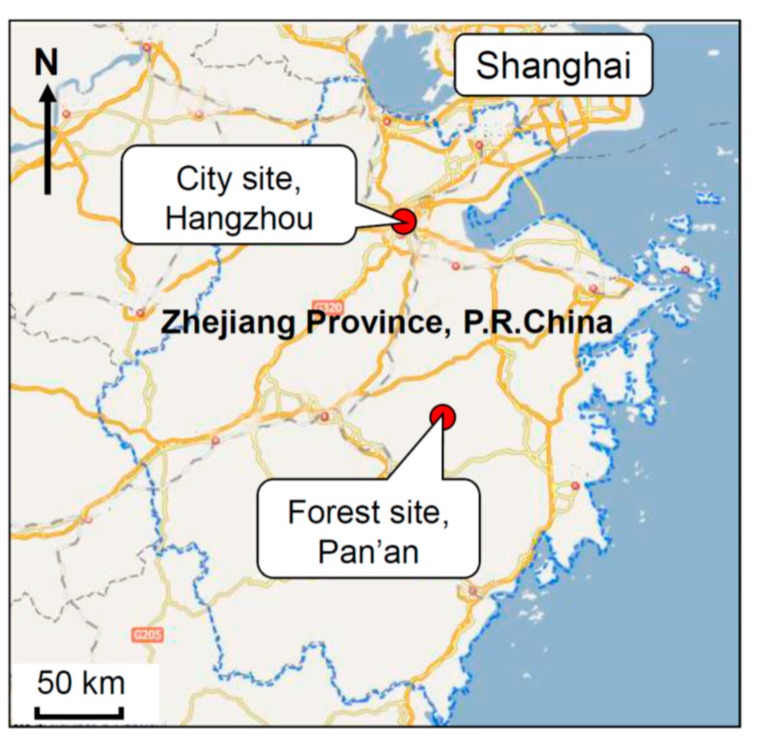
Location of the two experimental sites. Huangtan Forest Park is located in Pan’an County, Zhejiang Province, China. It is about 160 km from the urban experimental site, which is situated in the downtown area of Hangzhou, a city near Shanghai.

**Figure 2:**
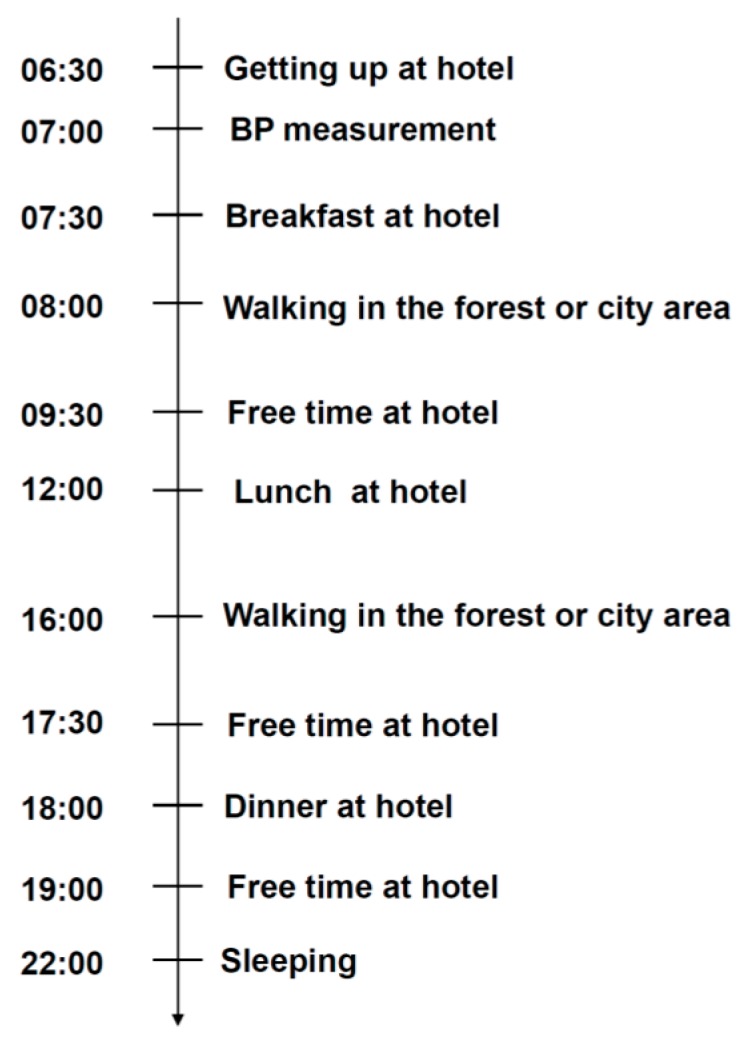
The experimental protocol for subjects exposed to the forest or city environment.

The experiment was conducted in a Forest Park named Huangtan located in Pan’an County (Zhejiang Province, China) from 20 to 24 August 2015. The covering area of the forest is about 8,799,000 m^2^ and the predominant species are pine, China fir, and bamboo. For comparison, a typical urban site located in the downtown area of Hangzhou was used as the control. The two experimental sites are shown on the map in Figure 1. Briefly, on the day before the experiment, all of the participants gathered at our hospital and were fully informed about the experimental procedure and signed informed consent from each participant was obtained. Then a routine physical examination for each participant was performed and blood was sampled in the morning before breakfast. After that, each participant was asked to fill in a questionnaire for the profile of mood states (POMS) test by paper and pencil. The participants were randomly divided into two groups and were sent to two hotels with similar accommodation conditions near the two indicated experimental sites, respectively. The distance between the forest site and the corresponding hotel was similar to that of the control city site and its nearby hotel which was about a 5–10 min walk. We rented two commercial buses equipped with drivers to transfer the two groups from our hospital to the respective experimental site, as well as for the return trip. To control for environmental conditions, the intake of all foods and physical activity were controlled, and smoking and alcoholic or caffeinated beverages were not allowed. The experimental schedule is shown in Figure 2. Of note, the subjects walked outdoors twice everyday during the experimental period, and each time they walked along a predetermined flat walking path in each area at an unhurried pace for about 1.5 h which caused an estimated 120 kcal consumption on average for each participant. As a consideration for the safety of the CHF patients, they were allowed the freedom to rest during the walk and were also accompanied by a nurse and a doctor. In their free time during their stay in the hotels, the subjects were allowed to do as they wished, such as reading, watching TV, or playing chess, while avoiding strenuous exercise and any stimulating activities. Additionally, the individual administration of drugs for each participant was carried out as usual. Blood samples were collected in the two hotels near each experimental site before breakfast on 24 August 2015 by the specialized technicians. Then they were asked to complete the POMS test for the second time and the experiment ended.

### 2.3. Bio-Indicators Determination

Blood samples were clotted for 30 min and centrifuged for 10 min at 3000× *g*, and the supernatant was collected as serum samples, which were directly tested in the clinical laboratory by technicians for high-sensitive-reactive protein (hCRP), or stored in small aliquots at −70 °C until use.

The bio-markers for heart failure BNP and NT-ProBNP, cardiovascular disease-related factors, including ET-1, and constituents of the renin-angiotensin system (RAS), such as renin, angiotensinogen (AGT), angiotensin II (Ang II), angiotensin II type 1 receptor (AT1), angiotensin II type 2 receptor (AT2), as well as pro-inflammatory cytokines, including interleukin-6 (IL-6), tumor necrosis factor α (TNF-α), were analyzed using commercially available enzyme-linked immunoassay (ELISA) kits (CUSABIO, Wuhan, China) according to the manufacturer’s protocols.

The activity for serum total SOD (T-SOD) and Lipid peroxidation reflected by malondialdehyde (MDA) levels were chosen as oxidative indicators. They were evaluated by using standard assay kits (Nanjing Jiancheng Bioengineering Institute, Nanjing, China) as described previously [19].

### 2.4. Profile of Mood States Evaluation

To assess the fluctuating active mood states of the participants, we used the standard version of the POMS test, which is a 65-item self-administered rating scale that measures six dimensions of mood (tension-anxiety, depression-dejection, fatigue-inertia, confusion-bewilderment, vigor-activity, and anger-hostility). We assessed the participants’ mood state changes before and after the experiment.

### 2.5. Air Quality Assessment

The air quality in the two sites was monitored during the experiment, simultaneously. The level of negative oxygen ions was detected by an air ion counter (KEC-900 Type, Shenzhen Yuan Hengtong Technology Co., Ltd., Shenzhen, China). The concentration of PM_2.5_ (particulate matter considered as mass defined by a size cutoff at 2.5 μm in aerodynamic diameter) was measured by a portable dust monitor (Dustmate Type, Beijing Liyang Tech Technology Co. Ltd., Beijing, China).

### 2.6. Data Analysis

The results are expressed as mean ± SD. Statistical analysis was performed using SPSS version 19.0 (obtained from SPSS China, Shanghai, China). Samples were initially analyzed using the Kolmogorov-Smirnov test and Levene’s test for, respectively, normality and homogeneity of variances. If the samples were closed to normal distribution and had homogeneous variance, the *t-*test was used for data comparison between the two groups. Otherwise, a non-parametric test (Mann-Whitney *U* test or Wilcoxon Signed Ranks test) was used for two independent or related samples. For the analysis of multi-group comparisons, the Kruskal-Wallis test was performed and a Dunn-Bonferroni test was used for post hoc comparisons. For the count data, the chi-squared test was used for data analysis. A *p*-value less than 0.05 was considered statistically significant.

## 3. Results

### 3.1. Effect of Forest Bathing on BNP and NT-ProBNP in Peripheral Blood

**Figure 3:**
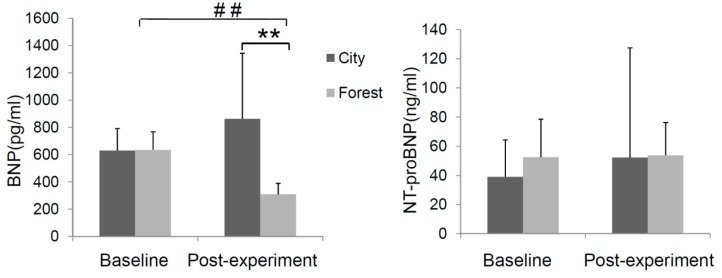
Effect of forest bathing on change of CHF biomarkers including brain natriuretic peptide (BNP) and NT-proBNP in the experimental subjects (forest group (*n* = 23) and control group (*n* = 10)). ** *p* < 0.01; ## *p* < 0.01, analyzed by the Kruskal-Wallis test, followed by the Dunn-Bonferroni test.

Circulating levels of B-type natriuretic peptide (BNP) are directly associated with cardiac hemodynamics and symptom severity in patients with HF and, therefore, serves as a marker of cardiac functional status. In patients with chronic stable HF, its circulating levels are positively associated with all-cause mortality [20]. N-terminal pro-BNP (NT-proBNP), a biomarker for HF related to BNP, was also detected. First of all, the effect of forest bathing on the CHF patients was assessed by circulating levels of BNP and NT-ProBNP. Obviously, as shown in Figure 3, the participants experiencing a four-day forest bathing trip showed a significant lower BNP levelcompared with that of the city group or itself baseline level before the experiment. While the BNP level of subjects exposed to the urban environment remained statistically unchanged. On the other hand, no significant changes of NT-ProBNP were observed in both of the groups before or after the experiment. However, the decreased BNP suggested a favorable effect of forest bathing on heart functional status.

### 3.2. Effect of Forest Bathing on Cardiovascular Disease-Associated Factors

**Figure 4:**
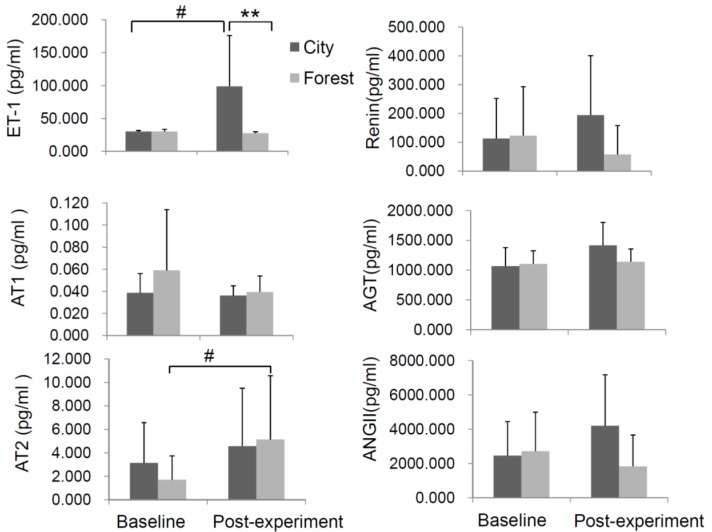
Effect of forest bathing on change for the endothelin-1 (ET-1) production and components of the renin-angiotensin system (RAS). Renin, angiotensinogen (AGT), angiotensin II (Ang II), angiotensin II type 1 receptor (AT1), and angiotensin II type 2 receptor (AT2) of subjects were evaluated before and after the experiment (forest group (*n* = 23) and control group (*n* = 10)). ** *p* < 0.01, # *p* < 0.05, analyzed by the Kruskal-Wallis test followed by the Dunn-Bonferroni test.

We next selected several cardiovascular disease-associated factors, including ET-1, and RAS constituents, such as renin, AGT, Ang II, AT1, and AT2, as these factors have been reported to be related to HF. A similar baseline level of these indicators was observed between the two groups before the experiment (Figure 4). At the end of the four-day experiment, a significantly lower level of ET-1 was observed in the forest group in comparison to that of city group (Figure 4, top-left panel). Meanwhile, the ET-1 level went up and the five RAS constituents remained significantly unchanged in the city group after the experiment. Of note, the levels of AT2, angiotensin type 2 receptor, which plays a protective role in the setting of chronic heart failure [21], increased in the forest group, while little alteration of this indicator was observed in the control city group (Figure 4, lower-left panel).

### 3.3. Effect of Forest Bathing on the Levels of Inflammatory Cytokines in the Serum

**Figure 5:**
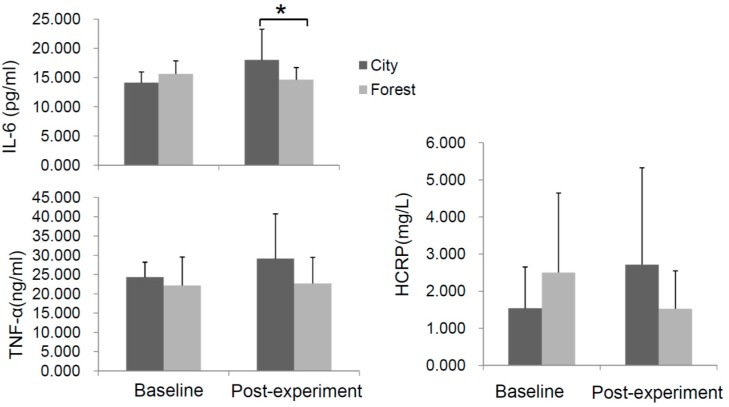
Effect of forest bathing on serum levels of pro-inflammatory indicators. Interleukin-6 (IL-6), tumor necrosis factor α (TNF-α), and C-reactive protein (CRP) of subjects were evaluated before and after the experiment in the forest bathing group (*n* = 23) as well as in the urban control group (*n* = 10). * *p* < 0.05, analyzed by the Kruskal-Wallis test followed by the Dunn-Bonferroni test.

We examined the levels of inflammatory proteins IL-6, TNF-α, and HCRP. As shown in Figure 5, after the experiment, the serum levels of IL-6 were significantly lower in the forest bathing group compared to the control group and the two other indicators (TNF-α and HCRP) in the forest group were ameliorative, compared with their baselines, although the changes were not significant (Figure 5). However, no significant alterations of these cytokines were observed in participants exposed to the urban environment.

### 3.4. Effect of Forest Bathing on Oxidative Stress

**Figure 6:**
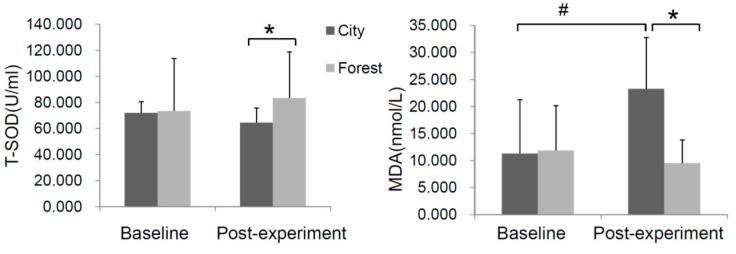
Effect of forest bathing on human oxidative stress alteration reflected by total superoxide dismutase (T-SOD) activity and malondialdehyde (MDA) level in the experimental subjects (forest group (*n* = 23) and control group (*n =* 10). * *p* < 0.05; # *p* < 0.05, analyzed by the Kruskal-Wallis test followed by the Dunn-Bonferroni test.

Subjects staying in the forest area showed a lower level of lipid peroxidation, as reflected by serum MDA, and a slightly higher level of T-SOD activity compared with the urban group (Figure 6). Of note, a significant increment of MDA was observed in subjects exposed to the urban environment. However, no obvious change in the activity of T-SOD was observed in the control group itself.

### 3.5. Effect of Forest Bathing on Mood State of Participants

**Figure 7:**
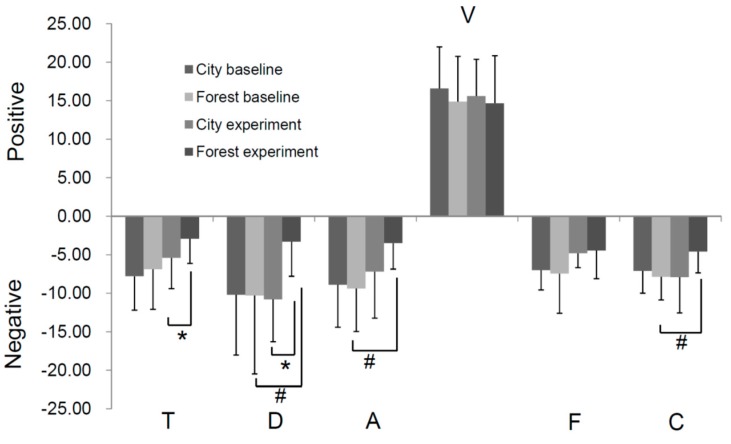
Profile of mood states (POMS) evaluation of subjects exposed to forest (*n* = 23) or city environments (*n =* 10). The standard version of POMS including negative subscales (T: tension-anxiety; D: depression-dejection; A: anger-hostility; F: fatigue-inertia; and C: confusion-bewilderment) and a positive subscale (V: vigor-activity) was used to evaluate the subjects’ mood changes before and after the experiment. * *p* < 0.05, # *p* < 0.05, analyzed by the Kruskal-Wallis test, followed by the Dunn-Bonferroni test.

It has been suggested that depressive mood could psychologically amplify the inflammatory response and deteriorate the process of cardiovascular diseases. We used the POMS standard version to evaluate the effect of the two different environmental stimuli on the psychological states of participants, as described previously [22]. As shown in Figure 7, after the experiment, a significant decrease was found in the forest group in four negative subscales: tension-anxiety (T), depression-dejection (D), anger-hostility (A), and confusion-bewilderment (C) compared with their baseline values or that of control group. However, no obvious change in any subscales was observed in the city group (Figure 7).

### 3.6. Air Quality Assessment

The air quality in both of the two experimental sites was monitored simultaneously. Overall, the air quality at the forest site was much better than that of the city site (Table 2). The level of negative ions in the daytime at the forest site was about 30-fold higher than that of the city site. The concentration of PM_2.5_ in forest sites (<10) was dramatically lower than that of city sites (>100).

## 4. Discussion

### 4.1. Main Founding of This Research

In recent decades, “Forest Therapy” has been increasingly recognized as a relaxation and stress management activity with demonstrated clinical efficacy. Forest bathing, a typical mode of “Forest Therapy”, has shown emerging evidence for its beneficial effects on human health, though the precise mechanism remains largely unknown. The data from the current study provided direct evidence of the salutary influence of forest bathing on elderly patients with chronic heart failure reflected by a lowered level of BNP and ET-1, accompanied by ameliorated inflammatory and oxidative status. Our findings further expanded the potential interventional efficacy of forest bathing on human health.

### 4.2. Relationship of the Beneficial Effect of Forest Bathing and the Environmental Parameters

It is strongly held that high levels of negative oxygen ions and some volatile organic compounds (VOCs) from volatile oils, such as phytoncide, in the forest both result in its promotion on human health [23,24]. However, a recent meta-analysis result showed that negative ionization was significantly associated with lower depression severity, with a stronger association observed at high levels of negative ion exposure (>2.7 × 10^6^ cm^−3^), which implies the beneficial effect of a high level of negative air ions on psychological status [25]. The average level of negative ions in the current forest site was about 10^5^ cm^−3^, but it is still much lower than 10^6^ cm^−3^, thus, it may slightly influence the psychological changes which further related to mental health and heart failure settings [26]. Additionally, the adverse effects of air pollution on cardiovascular health have been established in a series of major epidemiologic and observational studies [27,28,29]. In particular, PM_2.5_, known as the main component of haze, causes a serious environmental and public health problem in China, especially in large cities, e.g., Beijing. Recently, PM_2.5_ was reported to be highly related to specific microRNA expression which are associated with systemic inflammation, endothelial dysfunction, and atherosclerosis, indicating its potential regulation on gene expression and, thus, mechanistically known as a risk factor for human health [30,31]. However, the relationship between PM_2.5_ and heart failure remains controversial. Some studies indicated that there was no association between PM_2.5_ and BNP level, which is the biomarker of heart failure [32]. As anticipated, the level of PM_2.5_ was dramatically lower in the forest site compared to that in urban experimental site. On the other hand, the morbidity of cardiovascular diseases is reported to be related with lower ambient temperature (<10 °C) rather than higher temperature (>20 °C) [33,34]. In our current study, the ambient temperature in the two site was higher than 20 °C (Table 2), thus, we thought it was not the difference of ambient temperature that resulted in the effect of forest bathing on cardiovascular benefits. In addition, low atmospheric pressure and relative humidity was reported to be related to increased temperature-related mortality [35]. Thus, the physiological and psychological benefits of forest therapy on elder patients with CHF reflected by our results are, at least partly, related to the better ambient conditions. In general, our results were in line with previous studies showing that forest therapy reduces multiple physiological and psychological indices of stress in young adults [36,37].

### 4.3. Response of Inflammation and Oxidative Stress upon Forest Bathing

Oxidative stress and inflammatory activation are interrelated pathways that play important roles in the pathogenesis of heart failure [38]. Inflammation resulting from oxidative stress is involved in the complex syndrome of heart failure [39,40]. The role of oxidative stress in the connection between the various cardiovascular disease risk factors, including hear failure, and the clinical sequelae of disease associated with heart failure has been recognized according to the oxidative stress hypothesis [41]. The current experiment showed that forest bathing induced a decrease of oxidative stress levels accompanied by decreased inflammatory activation, which may partly result in its therapeutic effect on CHF patients.

### 4.4. Association of Mood State and Heart Failure

Previous studies of our group and others have demonstrated that forest bathing has positive effects on mental health mainly by reducing the stress levels [16,42]. In the present study, we also examined the psychological responses of forest bathing on CHF patients. POMS data indicated that forest bathing induced a decrease in the intensity of a negative mood state, such as tension, depression, and anger, while the city environment did not. The change in negative emotional states is important because it has been associated with increased mortality among individuals with heart failure [38]. Thus, our results indicate that forest bathing may be beneficial for CHF patients by regulating emotion.

### 4.5. Limitations of the Experiment

There are some limitations to our study. A limit of this study is the small sample size. Another limitation of our observation is that we only measured the indicators at specific time points. It would be interesting to use wearable devices for continuous monitoring of physiological changes in a future study. Further, the climatic factors also warrants investigation. We chose summer in this study as it is a hot season and the forest is suitable. It is not clear whether similar changes would be found in other seasons. Additionally, it would be better if we could have the chance to observe the long-term effect of forest bathing on CHF subjects.

## 5. Conclusions

To summarize, despite the small sample size, it is worth noting that forest bathing, even for a short time, induced a decrement of BNP, accompanied by dec reased levels of inflammatory cytokines and oxidative stress levels in CHF participants. Our data may pave the way for potential development of forest bathing as an effective adjunctive therapy on CHF patients. Thus, considering of health promotion, people are encouraged to move out from the urban crowded areas to the natural forest environment.

## Figures and Tables

**Table 1:** Clinical characteristics of the participants (mean ± SD or number (%)).

	Forest Group (*n* = 23)	City Group (*n* = 10)	*p* Value
Age (years)	72.86 ± 5.85	70.70 ± 3.68	0.341
Gender (M/F)	12/11	7/3	0.455 *
Hight (cm)	162.45 ± 8.56	163.50 ± 9.01	0.755
Weight (kg)	65.18 ± 7.40	65.20 ± 10.90	0.996
BMI (kg/m^2^)	24.72 ± 2.38	24.35 ± 3.45	0.722
SBP (mmHg)	141.4 ± 15.1	142.5 ± 15.7	0.849
DBP (mmHg)	80.6 ± 10.5	75.9 ± 14.1	0.296
HR (bmp)	76.2 ± 11.5	69.0 ± 12.2	0.113
New York Heart Association Class			0.845 *
I	1 (4.3%)	1 (10.0%)	
II	16 (69.6%)	6 (60.0%)	
III	6 (26.1%)	3 (30.0%)	

* Fisher’s Exact Test was used; others were analyzed by using the independent-samples *t-*test. BMI, body mass index; SBP, systolic blood pressure; DBP, diastolic blood pressure; HR, heart rate.

**Table 2:** Quantitative detection of air quality in the two experimental sites (mean ± SD).

	City Site	Forest Site	*p* Value
Negative ion (cm^−3^)	397.6 ± 230.8	11,575.0 ± 1399.7	0.003 *
PM_2.5_ (μg/m^3^)	160.1 ± 41.3	9.4 ± 4.1	<0.001
T (°C)	29.3 ± 4.3	25.2 ± 2.5	0.040
RH (%)	51.8 ± 15.2	77.3 ± 3.7	0.042

Note: * Mann-Whitney U test was used; others were analyzed by using the independent-samples *t*-test. PM_2.5_, particulate matter <2.5 μm in aerodynamic diameter; T, temperature; RH, relative humidity.

## Clinical Trial

This study is registered at www.chictr.org with identifier number ChiCTR-OOC-15006853.
